# Cross cultural adaptation and psychometric properties of the Bengali version of the Scale of Oral Health Outcomes for 5-year-old children (SOHO-5)

**DOI:** 10.1186/s12955-021-01681-4

**Published:** 2021-02-05

**Authors:** Masuma Pervin Mishu, Richard G. Watt, Anja Heilmann, Georgios Tsakos

**Affiliations:** 1 Department of Health Science, Faculty of Sciences, University of York, Heslington, York, YO10 5DD UK; 2 Research Department of Epidemiology and Public Health, University College London (UCL), 1-19 Torrington Place, London, WC1E 6BT UK

**Keywords:** Oral health related quality of life (OHRQoL), Scale of oral health outcomes for 5-year-old children (SOHO-5), Bengali, Children, Dental caries, Validity

## Abstract

**Background:**

The oral health related quality of life (OHRQoL) of children in Bangladesh has not yet been measured, as there is no validated OHRQoL measure for that population. The aim of this study was to cross-culturally adapt the child self-report and parental proxy report versions of the Scale of Oral Health Outcomes for 5-year-old children (SOHO-5) into Bengali and test their psychometric properties: face validity, construct validity (convergent and discriminant validity) and reliability (internal consistency and test–retest reliability), among 5–9-year-old children and their parents in Bangladesh and assess associations between dental caries/sepsis and OHRQoL in this population.

**Methods:**

The forward–backward translated Bengali SOHO-5 was piloted among 272 children and their parents to test its face validity. The questionnaire was administered to 788 children and their parents to evaluate its psychometric properties. Internal consistency of Bengali SOHO-5 was assessed using Cronbach’s alpha, and test–retest reliability was assessed using Kappa. Convergent and discriminant validity were assessed through nonparametric tests. The calculation of effect sizes and standard error of measurement facilitated the assessment of minimally important difference (MID) for SOHO-5. The associations of reporting an oral impact with caries and sepsis were assessed via logistic regression models.

**Results:**

Both child self-report and parental proxy report questionnaires showed good face validity. Cronbach’s alpha scores were 0.79 and 0.87 for child and parental questionnaire, respectively. A weighted Kappa score of 0.85 demonstrated test–retest reliability of child questionnaire. SOHO-5 scores were significantly associated with subjective oral health outcomes and discriminated clearly between different caries severity and sepsis groups. These differences were considerably higher than the MID. After adjusting for child’s age, sex, setting, maternal education and family income, the odds of reporting an oral impact were 2.25 (95% CI 1.98–2.56) and 4.44 (95% CI 3.14–6.28) times higher for each additional tooth with caries and sepsis, respectively.

**Conclusion:**

This study provided strong evidence supporting the validity and reliability of both versions of Bengali SOHO-5 as OHRQoL measures. Dental caries and sepsis were associated with poor OHRQoL in this population. The Bengali SOHO-5 is expected to be a useful outcome measure for research and clinical purposes in Bengali speaking child populations.

## Background

Globally, untreated caries is the most prevalent chronic condition for all ages [1]. There is consistent evidence that dental caries has an impact on the functional and emotional well-being of children, and is significantly associated with poorer oral health related quality of life (OHRQoL) [2–7]. In most low- and middle-income countries (LMICs) more than 90% of dental decay remains untreated due to limited access to oral health care [8, 9]. As a consequence, children suffer from dental pain and infection [10]. There is high prevalence of dental pain among children in LMICs [11–15], contributing to poor OHRQoL [16, 17].

Bangladesh is a lower-middle-income country in South East Asia, where oral health is still a neglected part of health policy [18]. While reliable national data for dental caries prevalence among Bangladeshi children are not available, a 2007 survey among 1700 rural and slum urban children showed high prevalence of untreated dental caries [19]. The estimation of the impact of chronic diseases on health related quality of life (HRQoL) is important in the context of developing and evaluating interventions, and distributing health care resources [20]. However, so far, no study has measured the OHRQoL among Bangladeshi children and there is no validated OHRQoL measurement scale for children in Bengali, the seventh most widely spoken language in the world. This highlights the need for a validated scale in Bengali to assess the OHRQoL in children.

A limited number of standardized and validated OHRQoL measures are available for young children [21–24]. In the past, many researchers considered young children to be unreliable respondents, preferring parental proxy ratings [25]. However, parental proxy reports alone may not represent the reality experienced by the child. Children and parents do not necessarily share similar views about OHRQoL [26]; and children themselves are in the best position to assess their symptoms and quality of life [27]. Clearly, the child’s age, developmental stage and social context need to be considered [28, 29] and there is evidence that 4–6-year-old children can report reliably on more concrete domains of their own general health and quality of life, including pain and dysfunction [30]. Therefore, children aged five years and older could be considered as the primary source of information regarding their OHRQoL [31–33] and parental perspectives could supplement the child’s evaluation [34]. Systematic reviews showed that by using age appropriate questionnaires, valid and reliable information can be obtained from children [35, 36].

The Scale of Oral Health Outcomes for 5 years old (SOHO-5), originally developed in 2012 in English and validated in a Scottish sample, includes questions for both children and parents with each containing seven items (details are in methods section) [24]. This measure has since been cross-culturally adapted for use in the Brazilian Portuguese [23, 37], Indonesian [38] Spanish [39] and Chinese languages [40]. The SOHO-5 is simple to use and has been shown to discriminate well between different caries severity groups, in relation to active caries lesions, pulp involvement and dental sepsis [24, 37].

Since there is no validated OHRQoL measurement scale for children in Bengali and dental caries in children is a common problem there, it is important to validate an OHRQoL measure among children in Bangladesh so that it can then be used as a subjective outcome in research and clinical practice. The SOHO-5 was chosen as a suitable OHRQoL measure for that age group and population, considering its simplicity and discriminant ability as well as very good overall psychometric performance in other cultures, some of which are in Asia. The aim of this study was to undertake a cross-cultural adaptation of the child self-report and parental proxy report versions of the SOHO-5 and test their psychometric properties: face validity, construct validity (convergent and discriminant validity) and reliability (internal consistency and test–retest reliability), among 5–9-year-old children and their parents in Dhaka, Bangladesh. In addition, we aimed to assess associations between dental caries/sepsis and OHRQoL in this population.

## Methods

Ethical approval for this study was obtained from the University College London Research Ethics Committee (5348/001) and the National Research Ethics Committee of Bangladesh (BMRC/NREC/2013–2016/879).

*Instruments* The SOHO-5 was used in this study. A detailed description of the SOHO-5 items and scoring for both child and parental questionnaires can be found elsewhere [24, 37]. In short, within the seven items of the child version of SOHO-5, children are asked whether they ever experienced difficulties due to their teeth on eating, drinking, speaking, playing, sleeping; and whether they avoided smiling due to pain in their teeth, and avoided smiling due to appearance of their teeth. Answers are reported using a 3-point scale (no, a little, a lot). The parental version refers to parental proxy reports on the children’s OHRQoL and has six items in common with the child version, but instead of the question on difficulty drinking asks whether the child’s oral health affected their self-confidence. Answer options are on a 5-point scale (not at all, a little, moderate, a lot, a great deal). The SOHO-5 scores are calculated as the sum of response codes, where higher scores represent a greater degree of oral impacts on the quality of life.

The adaptation of the Bengali SOHO-5 questionnaire was conducted with consideration given to the local culture, and in line with published standard guidelines [38–40]. Both the child and parental original English versions of the SOHO-5 questionnaires were forward and backward translated. First, two native Bengali speakers who are proficient in English worked independently to produce the forward translation into Bengali. Then a consensus forward translated version was established by reconciling the differences between the two aforementioned versions. The consensus version was then backward translated into English by an independent translator proficient in both languages (English and Bengali) who was not involved in the forward translation process. We further checked the resulting backward translation with the original English version and found no discrepancies, no incorrect response categories and no missing words/phrases.

The validity and reliability testing of the derived Bengali version of the SOHO-5 was conducted following standard procedures [44, 45].

In addition, children were asked questions about their oral health status. Satisfaction with oral health was assessed with the question “How happy are you with your teeth?” (answer options: very happy, a little happy, not happy). Presence of dental cavities was measured by asking “Do you have any holes in your teeth?” (answer options: no, yes, don’t know). Dental pain was assessed by asking “Have your teeth ever hurt you?” and “Do your teeth hurt now?” (answer options: no, a little and a lot). Parents were also asked about their child’s experience of dental pain through the questions: “Has your child ever had toothache in the past” and “Does your child currently have toothache” (answer options: no, yes). The parental questionnaire further included questions on socio-economic background (parental education, monthly gross family income).

### Face validity

*Participants* Face validity was tested in January 2015 in a pilot study in Dhaka, Bangladesh, that included 272 children (aged between 5 and 9 years) and their parents. Participants were recruited among children attending the Department of Paediatric Dentistry, Dhaka Dental College Hospital (the only Government dental hospital in Bangladesh), and from a school located in the vicinity of the hospital.

*Procedure* Three trained interviewers conducted face-to-face interviews with the children, using the child version of the SOHO-5 questionnaire. The parental version of the questionnaire was self-administered. Feedback from both children and parents demonstrated appropriateness and good comprehensibility. The resulting Bengali version of the SOHO-5 was used for the main study.

### Reliability and construct validity

*Participants* Participants were 788 children aged 5–9 years and their parents, who had not been involved in the pilot study. Children were randomly selected and recruited from the same hospital, and from three randomly selected schools located in the vicinity of the hospital. All 5–9-years-old children attending these schools and their parents were invited into the study. Children who had any systemic diseases or acute infections, fever or diarrhoea during the week preceding the data collection were excluded. Details of the selection processes are described elsewhere [46].

*Procedure* The Child version of the SOHO-5 questionnaire was interviewer-administered and the parental version of the questionnaire was self-administered. The SOHO-5 questionnaire was administered following the same procedures as in the pilot study. For the children recruited in the sample through their schools, the parental questionnaire was sent home with the child to be filled in by the parent. It was then returned to the school and collected by the study investigators. For the children recruited in the hospital, the parents completed the parental questionnaire during the time of their child’s dental examination.

The children underwent a non-invasive clinical dental examination to measure dental caries and sepsis, following the WHO Oral Health Surveys—Basic Methods 2013 [47]. The number of decayed, missing and filled teeth (DMFT/dmft index) and number of teeth with dental sepsis, assessed through the number of teeth with pulpal involvement, ulceration, fistula or abscess (PUFA/pufa index) [48], were recorded.

### Data analysis

For analytical purposes, dental caries was classified into four categories based on severity, where caries free children formed a distinct group and those with dental caries experience were divided into tertiles. In this study popultion, 26.8% children were caries free (dmft + DMFT = 0), 26.6% were in the low caries tertile (dmft + DMFT = 1–2), 29.9% were in the moderate caries tertile (dmft + DMFT = 3–5) and 16.6% were in the severe caries tertile (dmft + DMFT > 5). Dental sepsis was dichotomised into children with ‘no sepsis’ and those ‘having any sepsis’. The prevalence of dental sepsis (pufa + PUFA > 0) was 35.8%.

Internal consistency of the Bengali SOHO-5 was assessed using the standardised Cronbach’s alpha, as well as the inter-item and item-total score correlations [45]. Test–retest reliability was assessed for the child version of the questionnaire through the weighted Kappa statistic, by repeated administration of the questionnaire to a random sub-sample of 20 children. Construct validity can be assessed by linking the attribute of the measure to some other attribute by a hypothesis or construct [45]. Among the different ways to establish construct validity, convergent and discriminant validity were used in this study. As such, it is expected that the measure under test should correlate with other measures of this construct and can discriminate between different clinical groups [45]. To test convergent validity, associations between the SOHO-5 score and subjective measures of oral health status (satisfaction with teeth, self-reported cavities, current and past dental pain) were examined using nonparametric tests (Kruskal–Wallis for satisfaction with teeth and dental pain; and Wilcoxon rank-sum for self-reported cavities), as the SOHO-5 scores were not normally distributed. For the parental version, associations with current and past dental pain were assessed by Wilcoxon rank-sum test. To assess discriminant validity, first the associations between the overall SOHO-5 score and dental caries severity (4 groups) and dental sepsis (2 groups) were tested by Kruskal Wallis test and Wilcoxon rank-sum tests respectively. Furthermore, we estimated the minimally important difference (MID) for the child version [49], which allows to put the differences into context and facilitates their interpretation. For this, we considered differences in SOHO-5 scores between distinct dental caries and sepsis groups. We followed distribution-based approaches and reported the effect size (mean difference/standard deviation) and the standard error of measurement (standard deviation × √1-reliability).

Finally, logistic regressions were conducted to assess associations between dental caries/sepsis and the dichotomized SOHO-5 score (‘no oral impact’ versus ‘at least one oral impact’). The SOHO-5 score (outcome) was dichotomized because 43.4% children reported no oral impacts. Two separate sets of models were run, one with dental caries as the exposure and one with dental sepsis as the exposure. First, we estimated the unadjusted association and this was followed by a model adjusting for child’s age and sex, study setting (hospital versus school), maternal education, and family income. Data analysis was performed using STATA 13 [50]. Both unadjusted and adjusted odds ratios and their respective 95% confidence intervals (CI) were reported. All regression analyses were based on complete data as there were only 73 observations (9.3%) with missing data.

## Results

In total, 788 children participated in the study (237 recruited from the hospital and 551 from the schools) and 725 (92%) parents completed the parental questionnaires. Their mean age was 7.1 years (95% CI 7.05–7.19) and 388 (49.2%) were boys.

More than 56.6% of children reported at least one oral impact on their quality of life. Children’s SOHO-5 scores ranged from 0 to 14, with a median of 1 and a mean of 1.79 (95% CI 1.62–1.96). We found a similar overall prevalence from parental reports (58.5%), and scores ranged from 0 to 25 with a median of 1 and a mean of 3.45 (95% CI 3.08–3.81).

Difficulty eating was the most commonly reported impact, followed by difficulty sleeping (Table 1).

**Table 1:** Frequency of parent and child reported SOHO-5 items and dental pain (max. N = 725)

	Parent reported prevalence of impacts and dental pain (%)	Children reported prevalence and severity of impacts and dental pain		
		No (%)	Little (%)	A lot (%)
*SOHO-5 items*				
1. Difficulty eating	55.0	49.0	31.6	19.4
2. Difficulty drinking	Not assessed	85.3	10.5	4.2
3. Difficulty speaking	15.9	90.0	6.8	3.2
4. Difficulty playing	12.8	93.3	4.7	2.0
5. Avoid smiling (due to pain)	23.6	86.1	9.5	4.3
6. Avoid smiling (due to appearance)	11.6	91.4	6.5	2.2
7. Difficulty sleeping	31.3	71.9	16.9	11.2
8. Self confidence affected	21.7	Not assessed		
*Dental pain*				
Dental pain (ever)	52.8	44.9	33.6	21.4
Dental pain (current)	42.9	58.5	28.2	13.3

For the child version, inter-item correlations ranged from 0.18 to 0.53, with a mean inter-item correlation of 0.35 (Additional file 1: Table shown in supplementary file). Item-total correlations ranged from 0.33 to 0.39. The standardized Cronbach’s alpha was 0.79, indicating good internal consistency, and it did not increase when any of the items was deleted (except for one item: difficulty drinking) (Table 2).

**Table 2:** Internal consistency of children’s SOHO-5 scores (self-reported): Item total correlation coefficients and Cronbach’s Alpha

Items	Item total correlation	Cronbach’s Alpha if item deleted
Difficulty eating	0.35	0.77
Difficulty drinking	0.40	0.80
Difficulty speaking	0.34	0.75
Difficulty playing	0.34	0.75
Avoid smiling (due to pain)	0.33	0.74
Avoid smiling (due to appearance)	0.39	0.79
Difficulty sleeping	0.34	0.75
Standardized Cronbach’s alpha: 0.79		

The weighted Kappa statistic was 0.85 for the SOHO-5 scores of the child version, demonstrating excellent test–retest reliability. For the parental proxy reports, inter-item correlations ranged from 0.17 to 0.69 with a mean inter-item correlation of 0.48 (Additional file 1: Table shown in supplementary file). Item-total correlations ranged from 0.45 to 0.57. The standardized Cronbach’s alpha was 0.87 and it did not increase when any of the items was deleted (except for one item: avoid smiling due to pain) (Table 3).

**Table 3:** Internal consistency of children’s SOHO-5 scores (parent reported): Item total correlation coefficients and Cronbach’s Alpha

Items	Item total correlation	Cronbach’s Alpha if item deleted
Difficulty eating	0.46	0.84
Difficulty speaking	0.48	0.85
Difficulty playing	0.49	0.85
Avoid smiling (due to pain)	0.57	0.89
Avoid smiling (due to appearance)	0.45	0.83
Difficulty sleeping	0.46	0.83
Self confidence	0.46	0.83
Standardized Cronbach’s alpha: 0.87		

Children’s SOHO-5 scores were significantly associated with self-reported current dental pain, past experience of dental pain, satisfaction with oral health status and presence of cavities (Table 4). Children who reported severe current dental pain had significantly higher SOHO-5 scores (mean = 5.07; 95% CI 4.49, 5.64), indicating worse quality of life, than those without current dental pain (mean = 0.60; 95% CI 0.49, 0.72). Considerable differences in SOHO-5 were found in relation to the experience of dental pain. Furthermore, children who were very satisfied with their teeth had significantly lower SOHO-5 scores (mean score = 0.70; 95% CI 0.55, 0.85) compared to children who were not satisfied with their teeth (mean = 3.42; 95% CI 2.89, 3.95). Similarly, those who did not report dental cavities had significantly lower SOHO-5 scores (mean = 0.44; 95% CI 0.33, 0.56) than those who reported cavities (mean = 3.05; 95% CI 2.78, 3.32).

**Table 4:** Children’s SOHO-5 scores (self-reported): associations with subjective and clinical indicators of oral health (N = 725)

Oral health indicators		Percentage (%)		SOHO-5 scores Median (25–75 percentile)	SOHO-5 scores Mean (95% CI)	*p*
*Current dental pain*^a^						
No		58.5		0 (0–1)	0.60 (0.49, 0.72)	< 0.001
A little		28.2		2 (1–3)	2.70 (2.43, 2.98)	
A lot		13.3		4 (3–7)	5.07 (4.49, 5.64)	
*Ever had dental pain*^a^						
No		44.9		0 (0–0)	0.36 (0.23, 0.48)	< 0.001
A little		33.6		2 (1–3)	2.14 (1.91, 2.37)	
A lot		21.4		3 (1–6)	4.24 (3.82, 4.67)	
*Satisfaction with oral health*^a^						
Not satisfied		16.8		3 (1–6)	3.42 (2.89, 3.95)	< 0.001
A little satisfied		37.3		2 (1–4)	2.50 (2.22, 2.78)	
Very satisfied		45.9		0 (0–1)	0.70 (0.55, 0.85)	
*Presence*^c^ *of dental cavities*^b^						
No cavity		45.6		0 (0–0)	0.44 (0.33, 0.56)	< 0.001
Have cavity		47.5		2 (1–4)	3.05 (2.78, 3.32)	
*Dental caries*^a^						
dmft + DMFT = 0		26.8		0 (0–0)	0.06 (0.00, 0.12)	< 0.001
1–2		26.6		0 (0–0)	1.57 (1.28, 1.86)	
3–5		29.9		1 (0–2)	2.57 (2.26, 2.89)	
> 5		16.6		2 (1–3)	3.50 (3.02, 3.99)	
*Dental sepsis*^b^						
pufa + PUFA = 0		64.2		0 (0–1)	0.89 (0.74, 1.02)	< 0.001
pufa + PUFA > 0		35.8		3 (2–5)	3.42 (3.10, 3.74)	

^a^Kwallis test

^b^Wilcoxon rank-sum test

^c^Children that reported ‘don’t know’ (6.8%) in the question on the presence of dental cavities were excluded from this analysis

For discriminant validity, there were clear and significant differences in the SOHO-5 scores between different clinical dental caries and dental sepsis groups (Table 4). The mean SOHO-5 score was 0.06 (95% CI 0.00, 0.12) for children with no caries, being gradually higher for each group with higher caries severity and up to 3.50 (95% CI 3.02, 3.99) for the severe caries group. Children diagnosed with dental sepsis (pufa + PUFA > 0) had significantly higher SOHO-5 scores (mean = 3.42; 95% CI 3.10, 3.74) than children without dental sepsis (mean = 0.89; 95% CI 0.74, 1.02).

In order to put these differences in context, we calculated the respective effect sizes and the standard error of measurement. The mean difference of SOHO-5 scores between those with no caries and those grouped into the mild, moderate and severe caries groups were 1.51, 2.51 and 3.44 points respectively in SOHO-5 score. The respective effect sizes (ES) were 0.99, 1.43 and 1.69 (ES expressed in standard deviation units), which are considered large according to Cohen’s established criteria [51] and they were also gradually higher as groups with higher caries severity were compared to the no caries group. However, as ES are expressed in standard deviation units, they do not provide an indication of the actual meaningful difference in SOHO-5 score units. The respective standard error of measurement (SEM) estimates were 0.70, 0.81, and 0.93. SEM is expressed in actual units, ie SOHO-5 score, and indicates what can be considered as measurement error. The mean differences between the caries groups were much higher than the SEM, indicating a clinically meaningful difference in SOHO-5 scores between these distinct clinical groups. Similarly, the mean difference of SOHO-5 scores between children with and those without dental sepsis was 2.53, corresponding to an ES of 1.12, which is again indicative of a large difference. Furthermore, the aforementioned difference in SOHO-5 scores considerable exceeded the respective SEM of 1.04 that could be considered as the minimum difference in SOHO-5 scores that is clinically meaningful.

Significant associations were also observed for the parental version of SOHO-5 with having current dental pain, past experience of dental pain, and presence of cavities and dental sepsis (Table 5).

**Table 5:** Children’s SOHO-5 scores (parent reported): associations with subjective and clinical indicators of oral health (N = 725)

Oral health indicators	SOHO-5 scores median (25–75 percentile)	SOHO-5 scores mean (CI)	*p*
*Current dental pain*^a^			< 0.001
No	0 (0–1)	1.14 (0.85, 1.44)	
Yes	5 (2–10)	6.54 (5.93, 7.15)	
*Ever had dental pain*^a^			< 0.001
No	0 (0–0)	0.71 (0.46, 0.96)	
Yes	4 (2–9)	5.91 (5.35, 6.46)	
*Dental caries*^b^			
dmft + DMFT = 0	0 (0–0)	0.17 (0.06, 0.29)	< 0.001
1–2	1 (0–3)	2.54 (1.96, 3.11)	
3–5	3 (1–7)	4.67 (4.02, 5.32)	
> 5	6 (2–12)	7.77 (6.64, 8.91)	
*Dental sepsis*^a^			< 0.001
pufa + PUFA = 0	0 (0–2)	1.51 (1.22, 1.74)	
pufa + PUFA > 0	5 (2–11)	6.78 (6.07, 7.53)	

^a^Wilcoxon rank-sum test

^b^Kwallis test

The results of both unadjusted and adjusted logistic regression models showed significant and strong associations between dental caries experience and reporting oral impacts (Table 6). For each additional tooth with dental caries and dental sepsis, the odds of reporting oral impacts increased by 2.37 (95% CI 2.09, 2.69, P < 0.001) and 5.18 (95% CI 3.69, 7.26, P < 0.001) times respectively in the unadjusted model. After adjustment for child's age and sex, study setting (hospital versus school), maternal education, and family income as potential confounders, the odds of reporting an oral impact was 2.25 (95% CI 1.98–2.56) times for each additional tooth with dental caries and 4.44 (95% CI 3.14–6.28) times for each additional tooth with dental sepsis (P < 0.001 for both associations).

**Table 6:** Associations of child reported SOHO-5 scores with clinical indicators of oral health: unadjusted and adjusted logistic regression models (adjusted for age, sex, study setting, maternal education and family income) (N = 715)

Exposure	Unadjusted			Adjusted		
	OR	95% CI	*p*	OR	95% CI	*p*
Dental caries	2.37	2.09, 2.69	< 0.001	2.25	1.98, 2.56	< 0.001
Dental sepsis	5.18	3.69, 7.26	< 0.001	4.44	3.14, 6.28	< 0.001

## Discussion

This study was the first to employ the SOHO-5 scale to measure OHRQoL among Bangladeshi children. The results provided strong evidence of good psychometric properties for both the child self-report and the parental proxy report Bengali versions of the SOHO-5 (Additional file 2 for the Bengali SOHO-5 child and parental proxy questionnaires)..

A detailed and established procedure for cross-cultural adaptation was followed, which ensured that the true meaning of the questions was retained in the Bengali SOHO-5. Overall, both child and parental Bengali SOHO-5 versions demonstrated very good face validity. The sample consisted of 5–9-year-old children, while the original SOHO-5 study included only 5–6-year-olds. Older children may have comprehended the questions better and answered them more easily due to their more advanced cognitive development [52].

In terms of reliability, all inter-item correlations were positive and all item-total correlations were above the 0.20 arbitrary threshold [45]. In addition, none of the correlations was high enough to indicate redundancy of any item. Both child and parental SOHO-5 versions had a standardized Cronbach’s alpha clearly above the arbitrary threshold of 0.70 that indicates good internal consistency reliability. The value of alpha did not improve when any of the items were deleted, with one exception when it marginally increased. However, this is not sufficient justification to drop this item from the Bengali SOHO-5 as all other reliability results indicated excellent performance. The child SOHO-5 version also demonstrated excellent test–retest reliability.

The analysis of the construct validity indicated excellent performance, as the scores were significantly associated with different subjective measures of oral health. We also clearly demonstrated the ability of SOHO-5 to discriminate between different levels of caries severity. There was a graded association with worse OHRQoL for each more severe caries group. The associations between OHRQoL and clinical measures of caries and presence of dental sepsis were robust, as they remained significant and strong even after adjustment for a number of relevant confounders. Furthermore, these significant differences in mean SOHO-5 scores between different clinical oral health groups corresponded to large effect sizes and were clearly higher than the MID for this setting, thereby being also clinically meaningful [49]. Estimating the MID for SOHO-5 in this setting provided added value to our study, as it allowed us to put the aforementioned significant differences into context and show their clinical relevance. This approach should be standard practice in OHRQoL studies, but very few studies assessing psychometric properties have gone a step further and estimated the MID for the validated measures.

To our knowledge, the present study is the first to provide evidence of associations between dental caries/dental sepsis and self-reported OHRQoL in a Bangladeshi child population. These findings are in line with other studies from different countries, using different OHRQoL measures. Studies conducted in Brazil using the parent- reported Early Childhood Oral Health Impact Scale (ECOHIS) for preschool children found that higher ECOHIS scores were associated with higher caries levels [53–55], advanced carious lesions [56–58], pulpal involvement and dental infection [56], even after adjustment of socioeconomic factors [59–61]. Studies conducted in China [62], Hong Kong [63, 64] and India [65] also reported similar associations. Furthermore, the number of decayed and missing teeth [5], caries increment [66] and PUFA/pufa [67] were associated with higher Child Perception Questionnaire (CPQ 8–10 and CPQ 11–14) scores among children in Brazil and Egypt [68], and higher Child—Oral Impact on Daily Performance (Child-OIDP) scores among children in Thailand [69–71] and India [72].

The present study also has some limitations. The study sample mainly included children of middle to lower-middle socioeconomic status. Further research on assessing OHRQoL using a representative sample and including a wider range of socioeconomic groups [73] is recommended. The use of a validated self-report scale to measure the OHRQoL of young children should be encouraged in both clinical practice and research. Furthermore, as severe caries is highly prevalent in this population and strongly associated with worse OHRQoL, effective interventions should be developed to reduce prevalence and ensure early treatment of dental caries in children to improve their OHRQoL. In that context, it was not possible to assess the responsiveness of the SOHO-5 through this study due to its cross-sectional design. Future longitudinal studies using SOHO-5 should complement the assessment of its psychometric properties so that it is also tested as a potential outcome measure to evaluate the effectiveness of oral health interventions in young children.

## Conclusion

This study cross-culturally adapted the child self-report and parental proxy report versions of the SOHO-5 for Bengali children and provided strong evidence that both versions are valid and reliable measures of OHRQoL for 5–9-year-old children in this setting, demonstrating excellent reliability (internal consistency and test–retest reliability), face and construct (convergent and discriminant) validity. The Bengali SOHO-5 can prove useful for applications in research and clinical practice. Dental caries and dental sepsis were associated with poor OHRQoL in this population; therefore, future research should focus on interventions to address the caries burden and improve the OHRQoL among Bangladeshi children.

## Supplementary Information

**Additional file 1: Supplementary Table 1**. Internal consistency of children’s SOHO-5 scores (self-reported): inter-item correlation matrix. **Supplementary Table 2**. Internal consistency of children’s SOHO-5 scores (parent reported): inter-item correlation matrix.

**Additional file 2.** Bengali version of SOHO-5 child questionnaire and parental proxy questionnaire.

## Data Availability

The datasets generated and analysed in the current study are not publicly available as all the data used in the study were treated confidentially as the agreement with the University College London data protection; but the minimal dataset that would be necessary to interpret, replicate and build upon the findings reported in the article are available from the corresponding author on reasonable request.

## Abbreviations

OHRQoL: Oral health related quality of life
SOHO-5: Scale of Oral Health Outcomes for 5-year-old children
DMFT: Decayed, missing and filled teeth
PUFA: Pulpal involvement, ulceration, fistula and abscess
