# Supplementary material for: Cross cultural adaptation and psychometric properties of the Bengali version of the Scale of Oral Health Outcomes for 5-year-old children (SOHO-5)
Source: Health Qual Life Outcomes. 2021 Feb 5;19:46. doi: 10.1186/s12955-021-01681-4 (PMC7866745; doi:10.1186/s12955-021-01681-4)
Supplement: Supplementary file 1 — Additional file 1: Supplementary Table 1. Internal consistency of children’s SOHO-5 scores (self-reported): inter-item correlation matrix. Supplementary Table 2. Internal consistency of children’s SOHO-5 scores (parent reported): inter-item correlation matrix. [file 12955_2021_1681_MOESM1_ESM.docx]

Additional Files

File name: Additional file 1 (Supplementary Table 1 and 2)

File format: .docx

File name: Additional file 2 (Bengali translated SOHO-5 child questionnaire and parental proxy questionnaire)

File format: .pdf

Additional file 1 with Supplementary Table 1 and 2

Supplementary Table 1: Internal consistency of children’s SOHO-5 scores (self-reported): inter-item correlation matrix

|  | Difficulty eating | Difficulty drinking | Difficulty speaking | Difficulty playing | Avoid smiling (due to pain) | Avoid smiling  (due to appearance) | Difficulty sleeping |
| --- | --- | --- | --- | --- | --- | --- | --- |
| Difficulty eating | 1 |  |  |  |  |  |  |
| Difficulty drinking | 0.22 | 1 |  |  |  |  |  |
| Difficulty speaking | 0.37 | 0.18 | 1 |  |  |  |  |
| Difficulty playing | 0.33 | 0.27 | 0.53 | 1 |  |  |  |
| Avoid smiling  (due to pain) | 0.43 | 0 | 0.52 | 0.50 | 1 |  |  |
| Avoid smiling  (due to appearance) | 0.26 | 0.24 | 0.31 | 0.30 | 0.29 | 1 |  |
| Difficulty sleeping | 0.52 | 0.25 | 0.46 | 0.43 | 0.49 | 0.23 | 1 |

Mean inter-item correlation: 0.35 (P<0.001, Spearman’s correlation coefficient)

Supplementary Table 2: Internal consistency of children’s SOHO-5 scores (parent reported): inter-item correlation matrix

|  | Difficulty eating | Difficulty speaking | Difficulty playing | Avoid smiling (due to pain) | Avoid smiling  (due to appearance) | Difficulty sleeping | Self confidence |
| --- | --- | --- | --- | --- | --- | --- | --- |
| Difficulty eating | 1 |  |  |  |  |  |  |
| Difficulty speaking | 0.48 | 1 |  |  |  |  |  |
| Difficulty playing | 0.45 | 0.67 | 1 |  |  |  |  |
| Avoid smiling  (due to pain) | 0.24 | 0.25 | 0.17 | 1 |  |  |  |
| Avoid smiling  (due to appearance) | 0.66 | 0.50 | 0.49 | 0.39 | 1 |  |  |
| Difficulty sleeping | 0.66 | 0.50 | 0.52 | 0.24 | 0.63 | 1 |  |
| Self confidence | 0.64 | 0.47 | 0.50 | 0.26 | 0.69 | 0.67 | 1 |

Mean inter-item correlation: 0.48 (P<0.001, Spearman’s correlation coefficient)
