# Supplementary material for: Cross cultural adaptation and psychometric properties of the Bengali version of the Scale of Oral Health Outcomes for 5-year-old children (SOHO-5)
Source: Health Qual Life Outcomes. 2021 Feb 5;19:46. doi: 10.1186/s12955-021-01681-4 (PMC7866745; doi:10.1186/s12955-021-01681-4)
Supplement: Supplementary file 2 — Additional file 2. Bengali version of SOHO-5 child questionnaire and parental proxy questionnaire. [file 12955_2021_1681_MOESM2_ESM.pdf]

দৈনন্দিন কার্যাবলী সংক্রান্ত প্রশ্নমালা

১. দাঁতের সমস্যা বা দাঁত ব্যাথার কারণে কি তোমার খাবার খেতে সমস্যা হয়/কখনও হয়েছিল ?

- [ ] না
- [ ] অল্প/ সামান্য
- [ ] খুব বেশি

২. দাঁতের সমস্যা বা দাঁত ব্যাথার কারণে কি তোমার পানি/অন্য কোন পানীয় খেতে (পান করতে) সমস্যা হয়/কখনও হয়েছিল ?

- [ ] না
- [ ] অল্প/ সামান্য
- [ ] খুব বেশি

৩. তোমার দাঁতের কারণে কি কথা বলতে সমস্যা হয়/ কখনও হয়েছিল?

- [ ] না
- [ ] অল্প/ সামান্য
- [ ] খুব বেশি

৪. দাঁতের সমস্যা বা ব্যাথার কারণে কি তোমার খেলতে সমস্যা হয়/ কখনও হয়েছিল?

- [ ] না
- [ ] অল্প/ সামান্য
- [ ] খুব বেশি

৫. দাঁত ব্যাথার কারণে তুমি হাসতে পারো নাই/ হাসি খুশি ছিলেনা এমন কি কখনও হয়েছে?

- [ ] না
- [ ] মাঝে মধ্যে
- [ ] অনেক সময়

৬. দাঁত দেখতে কেমন লাগবে এই কথা ভেবে তুমি হাসে নাই এমন কি কখনও হয়েছে?

- [ ] না
- [ ] মাঝে মধ্যে
- [ ] অনেক সময়

৭. দাঁতের সমস্যা/ ব্যাথার কারণে কি তোমার রাত ঘুমাতে সমস্যা হয়/ কখনও হয়েছে ?

- [ ] না
- [ ] অল্প/ সামান্য
- [ ] খুব বেশি

উত্তর প্রদানের কার্ড

প্রতিটি প্রশ্নের জন্য আমি তোমাকে তিন ধরনের মুখের চেহারা দেখাব। তোমার যদি কোন সমস্যা না থাকে অথবা দাঁতে ব্যাথা বা কষ্ট না থাকে তাহলে হাসি মুখে চেহারাটি দেখাবে। যদি তোমার অল্প সমস্যা বা অল্প দাঁত ব্যাথা থাকে তাহলে মধ্যের চেহারাটি দেখাবে। আর যদি তোমার খুব বেশি সমস্যা থাকে অথবা খুব বেশি দাঁত ব্যাথা থাকে তাহলে দুঃখী মুখের চেহারাটি দেখাবে।

তোমার দাঁতে ব্যাথা আছে কিনা সেটা যে মুখটিতে বোঝা যাচ্ছে সেটা দেখাও।

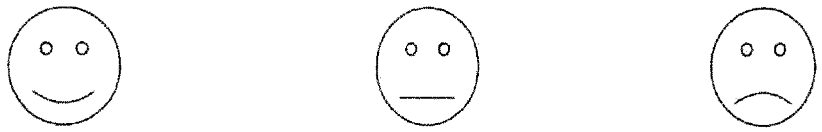

না	অল্প/ সামান্য	খুব বেশি
☐	☐	☐

আপনার সন্তানের দাঁতের অবস্থা এবং তার দৈনন্দিন জীবনে এর প্রভাব সংক্রান্ত প্রশ্নাবলী

১. আপনার সন্তানের জীবনে কখনো কি তার দাঁতের কারনে খাবার খেতে সমস্যা হয়েছে ?

☐ একদম নয়/ কখনো সমস্যা হয় নাই।
☐ সামান্য সমস্যা হয়েছে।
☐ মাঝারি ধরনের সমস্যা হয়েছে।
☐ অনেকে সমস্যা হয়েছে।
☐ প্রচন্ড/ মারাত্বক সমস্যা হয়েছে।

২. আপনার সন্তানের জীবনে কখনো কি তার দাঁতের কারনে কথা বলতে সমস্যা হয়েছে ?

☐ একদম নয়/ কখনো সমস্যা হয় নাই।
☐ সামান্য সমস্যা হয়েছে।
☐ মাঝারি ধরনের সমস্যা হয়েছে
☐ অনেকে সমস্যা হয়েছে।
☐ প্রচন্ড/ মারাত্বক সমস্যা হয়েছে।

৩. আপনার সন্তানের জীবনে কখনো কি তার দাঁতের কারণে স্বাভাবিক খেলাধুলা করতে অথবা স্কুলে যেতে সমস্যা হয়েছে ?

☐ একদম নয়/ কখনো সমস্যা হয় নাই।
☐ সামান্য সমস্যা হয়েছে।
☐ মাঝারি ধরনের সমস্যা হয়েছে।
☐ অনেকে সমস্যা হয়েছে।
☐ প্রচন্ড/ মারাত্বক সমস্যা হয়েছে।

৪. দাঁত দেখতে সুন্দর নয় অথবা দাঁতের অবস্থা ভাল নয় , সেটা ভেবে কি আপনার সন্তান কখনও তার স্বাভাবিক হাসি-খুশি থাকা থেকে বিরত থেকেছে ?

☐ কখনও বিরত থাকে নাই
☐ কদাচিত বিরত থাকে
☐ মাঝে মাঝে বিরত থাকে
☐ প্রায় সময় বিরত থাকে
☐ সব সময় বিরত থাকে

৫.দাঁতের সমস্যা অথবা দাঁত ব্যাথার কারনে কি সে কখনও তার স্বাভাবিক হাসি-খুশি থাকা থেকে বিরত থেকেছে?

☐ কখনও বিরত থাকে নাই
☐ কদাচিত বিরত থাকে
☐ মাঝে মাঝে বিরত থাকে
☐ প্রায় সময় বিরত থাকে
☐ সব সময় বিরত থাকে

৬. দাঁতের সমস্যার কারণে কি আপনার সন্তানের কখনও ঘুমের সমস্যা হয়েছে?

☐ একদম নয়/কখনও সমস্যা হয় নাই।
☐ সামান্য সমস্যা হয়েছে।
☐ মাঝারি ধরণের সমস্যা হয়েছে।
☐ অনেক সমস্যা হয়েছে।
☐ প্রচন্ড/মারাত্বক সমস্যা হয়েছে।

৭. দাঁতের সমস্যার কারণে কি আপনার সন্তানের আত্মবিশ্বাস ক্ষতিগ্রস্ত হয়েছে?

☐ একদম নয়
☐ সামান্য
☐ মাঝারি ধরণের
☐ বেশি
☐ খুব বেশি
